# Can detect the left ventricular contraction performance from pep using ecg r wave?

**DOI:** 10.1186/2197-425X-3-S1-A588

**Published:** 2015-10-01

**Authors:** K Yamashita, T Yatabe, T Tamura, H Tateiwa, T Kawano, M Yokoyama

**Affiliations:** Kochi Medical School, Anesthesiology and Intensive Care Medicine, Kochi, Japan

## Introduction

The onset of ventricular depolarization defines the start of the pre-ejection period (PEP), which is commonly used as an index of myocardial contractility. Although the fiducial point for this onset has traditionally been the onset of the Q wave of the electrocardiogram (qPEP), the Q wave is not visible in some patients. Therefore, other measurement points have also been used in the literature, including the peak of the R wave (rPEP). However, there has been little systematic examination of measurement issues associated with the selection of the fiducial point for ventricular activation.

## Objectives

The aim of this study is to evaluate rPEP for detecting the left ventricular contraction performance (dp/dt) compared with qPEP.

## Methods

After approval of the institutional animal ethics, six adult pigs weighting 40kg were anesthetized with an i.m. injection of ketamine, midazolam and atropine sulfate. Anesthesia was maintained isoflurane in oxygen, ketamine and vecronium bromide. Intubation was undertaken. Mechanical ventilation was started with 10ml/kg in tidal volume. An electrocardiography, ascending aortic pressure and left ventricular pressure were monitored by polygraph-system (RMT-1000, Nihon Kohden, Tokyo, Japan). A pulmonary artery catheter was inserted by pressure curve visualization. CO was measured by thermodilution method (774HF75, Edwards Lifescience LLC, Irvine, CA, USA). Spearman's rank correlation coefficient and Blant-Altman analysis was used to evaluate the ability for the left cardiac contraction performance.

## Results

Two-hundred points are simultaneously measured respectively. rPEP has a good correlation to qPEP (rPEP = 35.1 + 0.82 qPEP; rs = 0.84, P < 0.0001). The Bias and standard error between rPEP and qPEP are 16.3 +/- 0.8 (14.8 to 17.8; 95%CI). rPEP also has a good ability for estimating the left cardiac contraction performance (dp/dt = 2807.6 - 14.3 rPEP; rs = 0.68, p < 0.0001).

## Conclusions

rPEP is correlated well with qPEP. Therefore, rPEP could use an alternative parameter for left cardiac contraction performance instead of qPEP under various clinical setting.

## Grant Acknowledgment

This work was supported by Grant-in-Aid for Scientific Research (C) 26462361.
